# Performance of rapid rk39 tests for the diagnosis of visceral leishmaniasis in Ethiopia: a systematic review and meta-analysis

**DOI:** 10.1186/s12879-021-06826-w

**Published:** 2021-11-17

**Authors:** Dawit Gebreegziabiher Hagos, Henk D. F. H. Schallig, Yazezew K. Kiros, Mahmud Abdulkadir, Dawit Wolday

**Affiliations:** 1 College of Health Sciences, School of Medicine, Department of Medical Microbiology and Immunology, Mekelle University, Mekelle, Ethiopia; 2 College of Health Sciences, School of Medicine, Department of Internal Medicine, Mekelle University, Mekelle, Ethiopia; 3 College of Health Sciences, School of Medicine, Mekelle University, Mekelle, Ethiopia; 4 Academic Medical Centre at the University of Amsterdam, Department of Medical Microbiology, Experimental Parasitology Unit, Amsterdam University Medical Centres, Meibergdreef 9, 1105 AZ Amsterdam, The Netherlands

**Keywords:** VL, rk39, Sensitivity, Specificity, Ethiopia

## Abstract

**Background:**

Visceral Leishmaniasis (VL) is a severely neglected disease affecting millions of people with high mortality if left untreated. In Ethiopia, the primary laboratory diagnosis of VL is by using an antigen from a 39-amino acid sequence repeat of a kinesin-related (rK39) of leishmania donovani complex (*L. donovani*), rapid diagnostic tests (RDT). Different rk39 RDT brands are available with very variable performance and studies from Ethiopia showed a very wide range of sensitivity and specificity. Therefore, a systematic review and meta-analysis were conducted to determine the pooled sensitivity and specificity of rk39 RDT in Ethiopia.

**Method:**

PUBMED, EMBASE, and other sources were searched using predefined search terms to retrieve all relevant articles from 2007 to 2020. Heterogeneity was assessed by visually inspecting summary receiver operating curves (SROC), Spearman correlation coefficient (r_s_), Cochran Q test statistics, inconsistency square (I^2^) and subgroup analysis. The presence and statistical significance of publication bias were assessed by Egger's test at p < 0.05, and all the measurements showed the presence of considerable heterogeneity. Quality assessment of diagnostic accuracy studies (QUADAS-2) checklists was used to check the qualities of the study.

**Results:**

A total of 664 articles were retrieved, and of this 12 articles were included in the meta-analysis. Overall pooled sensitivity and specificity of the rk39 RDT to diagnose VL in Ethiopia were 88.0% (95% CI 86.0% to 89.0%) and 84.0% (95% CI 82.0% to 86.0%), respectively. The sensitivity and specificity of the rk39 RDT commercial test kits were DiaMed: 86.9% (95% CI 84.3% to 89.1%) and 82.2% (95% CI 79.3% to 85.0%), and InBios: 80.0% (95% CI 77.0% to 82.8%) and 97.4% (95% CI 95.0% to 98.8%), respectively.

**Conclusion:**

Referring to our result, rk39 RDT considered an essential rapid diagnostic test for VL diagnosis. Besides to the diagnostic accuracy, the features such as easy to perform, quick (10–20 min), cheap, equipment-free, electric and cold chain free, and result reproducibility, rk39 RDT is advisable to remains in practice as a diagnostic test at least in the remote VL endemic localities till a better test will come.

## Background

**Fig. 1:**
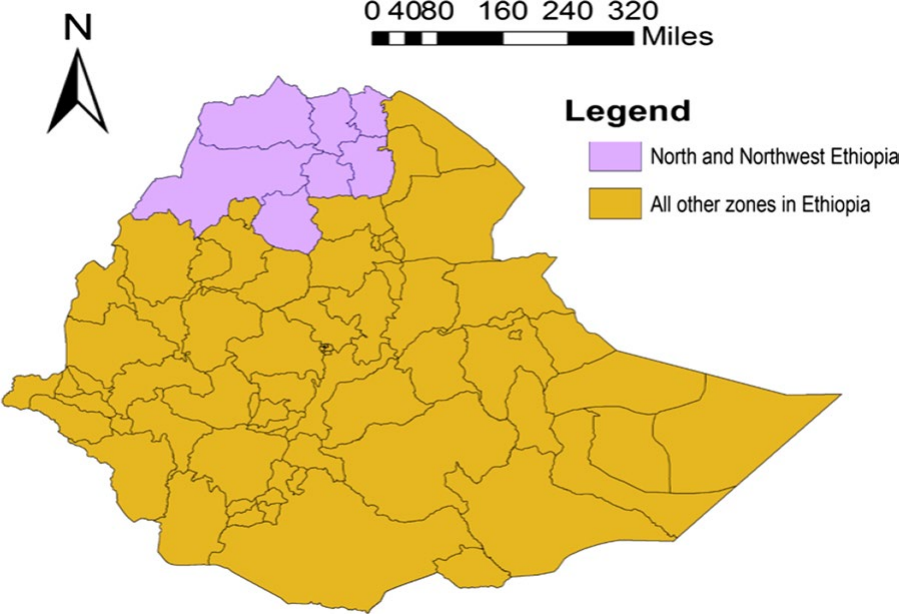
A map showing the north and northwest, and remaining other areas of Ethiopia

Visceral leishmaniasis (VL), or kala-azar, is a neglected tropical parasitic disease caused by a group of intracellular hemoflagellate protozoans of the genus *Leishmania* and transmitted via the bite of infected female *Phlebotomine* sandflies [1, 2]. Over 90% of the global VL burden is attributed to six less developed countries: Bangladesh, Brazil, India, Ethiopia, Sudan, and South Sudan [3–5]. Ethiopia ranks third among the world's most VL-affected countries and around 3.2 million people in the country are at risk of contracting the disease [6, 7]. The northern and northwest parts of the country have the highest burden, (Fig. 1), which accounts for nearly 30–40% of the total number of Ethiopian VL patients [8]. It is estimated that about 30% of the VL patients are also malnourished and co-infected with HIV, especially in the northern region of Ethiopia [9].

As VL is a deadly disease, timely and accurate diagnosis is important to install appropriate treatment [5]. The diagnosis is based on the combination of clinical signs and symptoms with laboratory confirmation [10]. The laboratory confirmation is done by demonstrating *Leishmania* parasites in microscopic preparations from splenic, or bone marrow, or lymph node aspirates, which is considered to be the gold standard test [11]. However, the low sensitivity combined with the invasive and risky sample collection procedures deterred the implementation of microscopy in the remote endemic areas of Ethiopia [12]. To circumvent the drawbacks of direct parasitological methods, serology has now been put in place in many regions of the country for the diagnosis of VL [13]. The direct agglutination test (DAT) is a pioneer serological test based on the agglutination of a *Leishmania* promastigote antigen preparation with specific antibodies in patient serum, which result can be interpreted without any reading aid. The DAT is robust, as the freeze-dried antigen with proven high sensitivity and specificity in all VL endemic regions around the world at an affordable price [14]. The drawback of DAT is relatively long overnight incubation and RDTs have been proposed as alternatives. In particular, Rk39 RDT detects antibodies against the 39-amino acid repeat antigens encoded by a kinesin-related gene of the amastigotes stage of the *Leishmania infantum* [15, 16], is considered to be a good alternative. The rK39 RDTs are simple to perform, cost-effective, stable at room temperature, and rapid. These immunochromatographic tests are currently widely implemented for the diagnosis of VL in resource-limited countries like Ethiopia [17, 18]. However, limitations of rk39 RDTs are variable specificity, inability to differentiate between current and past infection, not being suitable for treatment effectiveness monitoring [19].

Studies performed in Ethiopia, which evaluated the diagnostic accuracy of rk39 RDT, showed a large variation with sensitivities, ranging from 27.8% [20] to 98.3% [21]. Similarly, the specificities also showed a huge variation from 27.8% [20] to 98.2% [22]. Despite these variations, Ethiopia does not have nationwide and regional data that showed the diagnostic accuracy of the rk39 test. Therefore, this review and meta-analysis aimed to determine the pooled national sensitivity and specificity of the rk39 test and to assess if there is a difference between the different regions of the country.

## Method and materials

### Study design

A systematic review and meta-analysis was performed following the Cochran library recommendations for determining diagnostic test accuracy to assess the nationwide pooled sensitivity and specificity of the rk39 RDTs produced by InBios International Inc. (Seattle, WA, USA) or DiaMed-IT Leish®, DiaMed AG, Cressiersur-Morat, Switzerland, DiaMed Bio-RAD France, and Kalazar Detect® (InBios International, USA, and onsite Leishmania Ab Rapid Test (CTK Biotech, USA).

### Inclusion criteria

Original articles that determined the diagnostic accuracy of the index tests (rk39 RDT) for diagnosis of VL using human specimen, have a reference/s test, presence of the actual number of true positive, true negative, false-positive, and false-negative, and has a clear classification of study subjects into VL patients and controls were included in the systematic review and meta-analysis.

### Exclusion criteria

Articles were excluded if not clearly define the reference test and patient and control groups. Studies that used non-human specimens were also excluded.

### Search strategy

Electronic search in MEDLINE (via PUBMED), EMBASE, and Google Scholar was performed to retrieve articles evaluating the diagnostic performance of rk39 RDT for VL published from 2004 to 2020. To include more articles, we performed searching in other databases such as web of Science and SCOPUS using searching terms like evaluation, performance, validation, sensitivity, and specificity of rk39, Visceral Leishmania, VL, kala-azar, *L. donovani, L. infantum,* and Ethiopia. Duplicates cleaning was performed by using *EndNote X8* software. Primarily selection was done by reading titles and abstracts. Subsequently, articles found eligible by initial selection were further screened by reading the full text. The initial screening was independently performed by Dawit G. Hagos and Dr. Dawit Wolday. Discrepancies were resolved by discussion among the members of the team. To retrieve the relevant articles, the following search terms were included: PUBMED:- *((((((("leishmaniasis visceral"[MeSH Terms] OR "Kala-azar"[tiab]) AND "L. infantum"[tiab]) OR "L. donovani"[tiab]) OR "L. chagasi"[tiab]) AND (("diagnostic performance"[tiab] OR "rk39"[tiab]) OR "validation"[tiab])) AND "Ethiopia"[tiab]*. The search terms for EMBASE were: *(('visceral leishmaniasis'/exp OR 'kala azar'/exp OR 'l. donovani' OR 'l. infantum') AND 'performance of rk39' OR 'evaluation of rk39' OR 'evaluation'/exp) AND Ethiopia.* Furthermore*,* to search for unpublished manuscripts, institutions specific libraries such as Ethiopian University websites were searched. On top of this, references of the included articles were checked for cross-reference. Neither language nor time restriction was applied.

### Data analysis

Data were first extracted into a Microsoft Excel spreadsheet and sensitivity, specificity, positive and negative likelihood ratios, and diagnostic odds ratio were calculated using Meta-DiSc software, developed by *Clinical Biostatistics Unit—Hospital Ramón y Cajal, Hospital University of Madrid, Spain* and results were presented into a summary table and forest plot. Besides, the summary of receiver operating characteristic (ROC) plot was also generated, using sensitivity on the Y-axis and 1-specificity on the X-axis, which classifies the patient into VL and non-VL [23]. Heterogeneity was assessed by Inconsistent square (I^2^), visually inspecting SROC curves, subgroup analysis, and Spearman correlation coefficient (r_s_), where r_s_ > 0.6 indicates the presence of heterogeneity [24]. Inconsistency square (I^2^) statistics, categorised heterogeneity in to low (I^2^ < 25%), moderate (I^2^ = 25–75%) and high (I^2^ > 75%) [25]. Cochran Q test statistics (Chi-square) was also performed to explore the presence of heterogeneity. When the chi-square statistics p-value is < *0.05*, then the heterogeneity present is significant [26]. Publication bias was ascertained by performing Egger’s test p-value and publication bias *p* < *0.05* was considered as statistically significant. The quality of the selected articles was evaluated using QUADAS-2 checklists [27, 28].

## Results

**Fig. 2:**
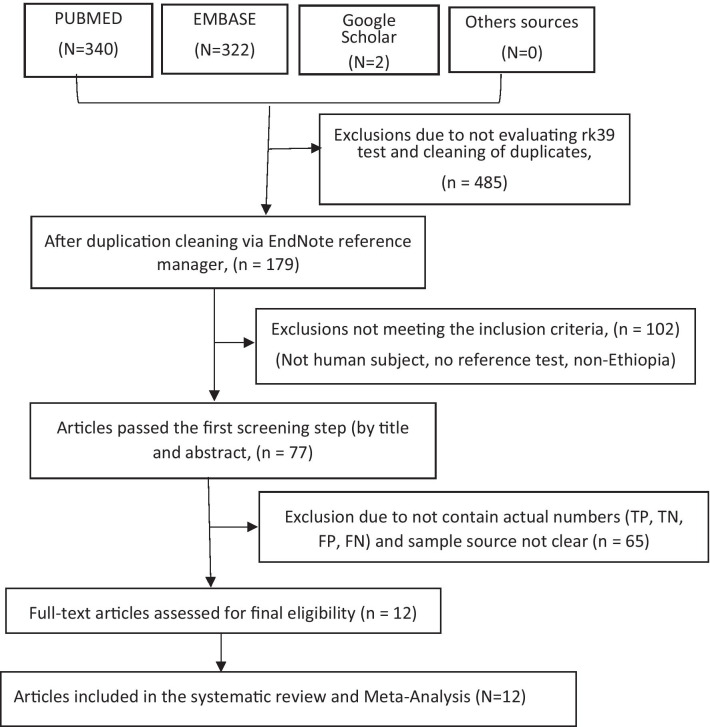
PRISMA Workflow Diagram for the article’s selection process

Using the search terms, 340, 322, and 2 articles from PUBMED, EMBASE, and Google scholar online databases respectively were retrieved. Besides, the references of all the included articles were checked for cross-referencing and obtained null. Moreover, other databases such as web of Science, SciELO and SCOPUS were also searched but no additional eligible articles were retrieved. No eligible gray or unpublished articles were obtained by personal communications and by searching specific Ethiopian University libraries. After duplicates were removed using *the EndNote X8 reference manager,* an initial selection process was performed by reading titles and abstracts and end up with 77 articles. Further screening was also done by reading the full-text length and finally, 12 articles were included in the meta-analysis (Fig. 2). From the 12 articles, 2240 data were extracted and included in the final random-effects models of meta-analysis.

**Fig. 3:**
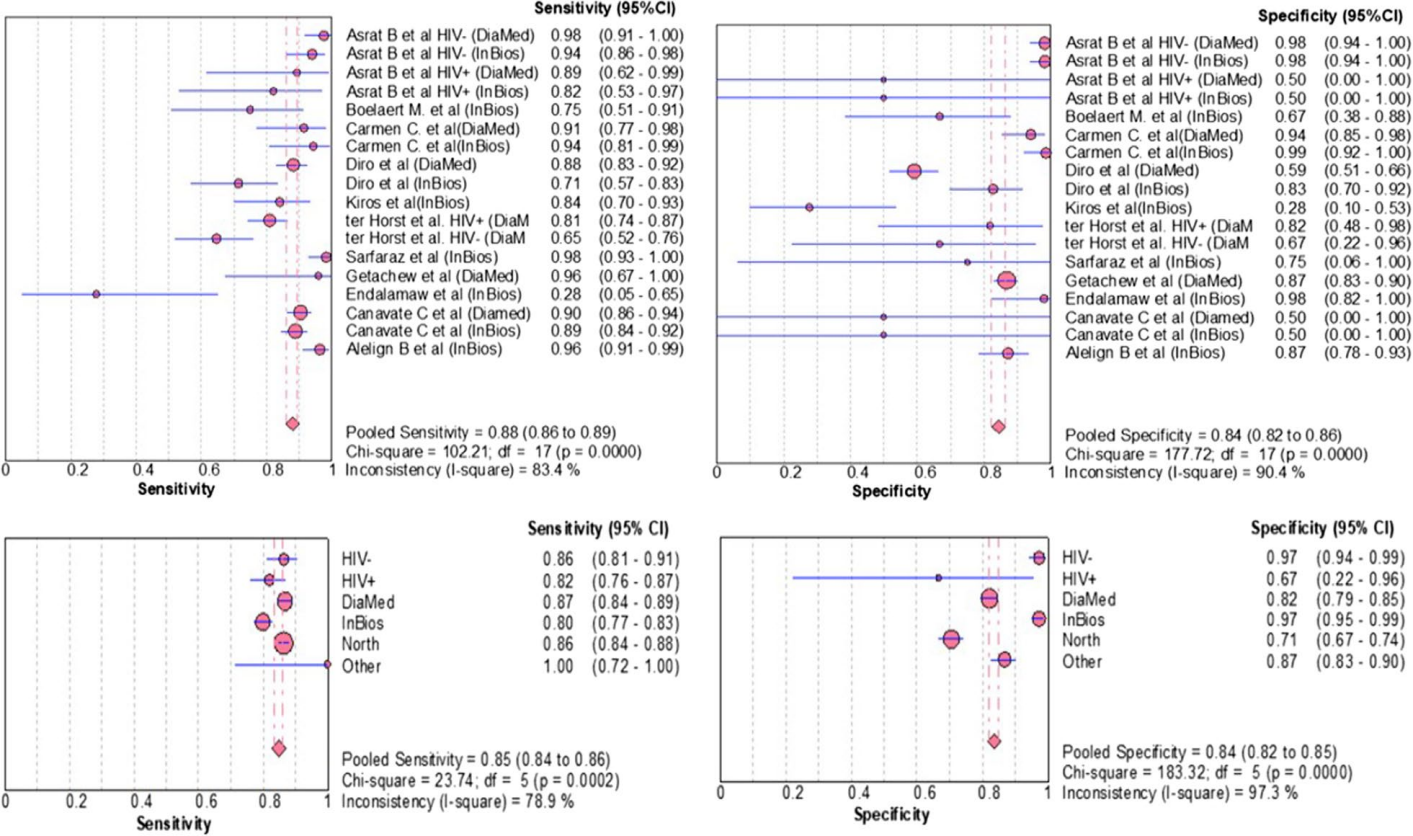
Estimates of sensitivity and specificity of rK39-based RDTs for the serodiagnosis of VL in Ethiopian studies

The systematic review showed a sensitivity of the rk39 RDT for VL diagnosis in Ethiopia ranged from 27.8% [9] to 98.3% [21]. Similarly, specificity was varied from 27.8% [20] to 98.5% [17], (Table 1). The pooled sensitivity and specificity of the included articles were also computed and the overall pooled sensitivity and specificity of the rk39 RDTs for VL diagnosis in the Ethiopian studies were 88.0% (95% CI 86.0% to 89.0%) and 84.0% (95% CI 82.0% to 86.0%), respectively, (Fig. 3).

**Table 1:** Compiled eligible studies included in the meta-analysis of rk39 in Ethiopia

Author	Year	TP	FN	FP	TN	Ref. test	Sample type	Control group	Sens (95% CI)	Spec (95% CI)
Asrat et al. HIV- (DiaMed)	2014	80	2	2	109	DAT	Serum	Healthy non-endemic, Healthy endemic & Other disease control	0.976 (0.98–0.99)	0.982 (0.93–0.99)
Asrat et al. HIV- (InBios)	2014	77	5	2	109	DAT	Serum	Healthy non-endemic, Healthy endemic & Other disease control	0.939 (0.88–0.97)	0.982 (0.98–0.93)
Asrat et al. HIV + (DiaMed)	2014	12	1	0	0	DAT	Serum	Healthy non-endemic, Healthy endemic & Other disease control	0.893 (0.60–0.98)	0.500 (0.02–0.98)
Asrat et al. HIV + (InBios)	2014	11	2	0	0	DAT	Serum	Healthy non-endemic & Healthy endemic & Other disease control	0.821 (0.54–0.95)	0.500 (0.02–0.98)
Boelaert et al. (InBios)	2008	15	5	5	10	DAT	Serum	Endemic patient control	0.750 (0.52–0.89)	0.667 (0.41–0.85)
Carmen et al. (DiaMed)	2011	32	3	4	63	Microscopy	Serum	Healthy endemic control	0.914 (0.77–0.97)	0.940 (0.85–0.98)
Carmen et al. (InBios)	2011	33	2	1	66	Microscopy	Serum	Health endemic control	0.943 (0.80–0.99)	0.985 (0.90–0.99)
Diro et al. (DiaMed)	2015	165	22	72	104	Microscopy&NNN	Serum	Endemic patient control	0.882 (0.83–0.92)	0.591 (0.52–0.66)
Diro et al. (InBios)	2007	35	14	9	43	Microscopy&NNN	Serum	Endemic patient control	0.714 (0.57–0.82)	0.827 (0.70–0.91)
Kiros et al.(InBios)	2017	37	7	13	5	Microscopy	Serum	Endemic patient control	0.841 (0.70–0.92)	0.278 (0.12–0.52)
ter Horst et al. HIV + (DiaMed)	2009	136	32	2	9	Micoscopy	Dried blood spot(DBS)	Other diseased patients	0.810 (0.74–0.86)	0.818 (0.49–0.95)
ter Horst et al. HIV- (DiaMed)	2009	42	23	2	4	Microscopy	DBS	Other diseased patients	0.646 (0.52–0.75)	0.667 (0.27–0.92)
Sarfaraz et al. (InBios)	2019	85	1	0	0	Microscopy	Serum	Other diseased patient & healthy endemic	0.983 (0.92–0.99)	0.500 (0.02–0.98)
Getachew et al. (DiaMed)	2019	11	0	46	304	*ELISA	Whole blood	Endemic Patient control	0.958 (0.58–0.99)	0.868 (0.82–0.90)
Endalamaw et al. (InBios)	2012	2	6	0	23	DAT	DBS	Endemic patient control	0.278 (0.08–0.62)	0.979 (0.74–0.99)
Canavate et al. (Diamed)	2011	223	23	0	0	PCR	Serum	Healthy endemic	0.905 (0.86–0.94)	0.500 (0.02–0.98)
Canavate et al. (Inbios)	2011	219	27	0	0	PCR	Serum	Healthy endemic	0.889 (0.84–0.92)	0.500 (0.02–0.98)
Alelign et al. (InBios)	2020	106	4	11	75	Micoscopy	Serum	Other diseased patient & healthy control	0.96 (0.91–0.99)	0.87 (0.78–0.93)

A large deviation of sensitivity and specificity among the individual studies and from pooled was observed. To explore the presence of heterogeneity, different methods such as I^2^, Chi-square, r_s_, visual inspection of SROC, and subgroup analysis were employed. The I^2^ statistics demonstrated considerable heterogeneity: sensitivities (I^2^ = 83.4) and specificity (I^2^ = 90.4%). The Chi-square p-value was very low (*P* = *0.0000*) and hence the heterogeneity present was significant, not by chance [26].

In addition, r_s_ was computed between sensitivity (logit of the true positive rate) and specificity (logit of the false positive rate) for rk39 RDT [24]. In our analysis, r_s_ = 0.699, P = 0.043, suggesting a significant threshold effect, which in turn explained there was a significant heterogeneity [29].

**Fig. 4:**
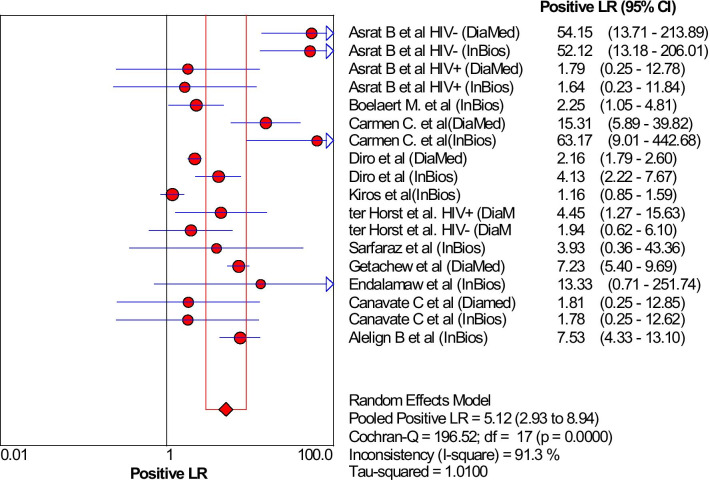
Estimates of positive likelihood ratio of rK39 for the diagnosis of VL in Ethiopian studies

By performing the subgroup analysis, between study heterogeneity was determined, and only these using different reference test was found a cause for heterogeneity (I^2^ reduced from 92.1 to 77.6). Neither the different Ethiopian regions nor type of commercial kits were the found the cause for heterogeneity. The sensitivity and specificity of the rk39 in north Ethiopia, a region with highest VL prevalence, were found lower: 86.0% (95% CI 84.0% to 88.0%) and 71.0% (95% CI 75.0% to 92.0%) than the rest: 100.0% (95% CI 72.0% to 100.0%) and 87.0% (95% CI 83.0% to 90.0%), respectively. Furthermore, these studies that used InBios International Inc. kit had higher sensitivities and specificities (89.2%, 95% CI 86.7% to 91.4%, I^2^ = 84.3% and 88.6%, 95% CI 84.9% to 91.6%, I^2^ = 87.7%) than DiaMed-IT(86.7%, 95% CI 84.2% to 89.0%, I^2^ = 83.5% and 82.1%, 95% CI 79.1% to 84.8%, I^2^ = 92.7) respectively. The rk39 RDT that uses microscopy and/or NNN culture as reference test had lower sensitivity and specificity (84.4%, 95% CI 81.4% to 87.1%, I^2^ = 85.6% and 74.1%, 95% CI 69.5% to 78.3%, I^2^ = 92.1%) than those used serology (90.7%, 95% CI 88.5% to 92.6%, I^2^ = 77.6% and 90.2%, 95% CI 87.7% to 92.3%, I^2^ = 78.2%). The likelihood ratio of the rk39 was also determined. The overall positive likelihood ratio (LR) were 5.12 (95% CI 2.93% to 8.94%), (Fig. 4).

**Fig. 5:**
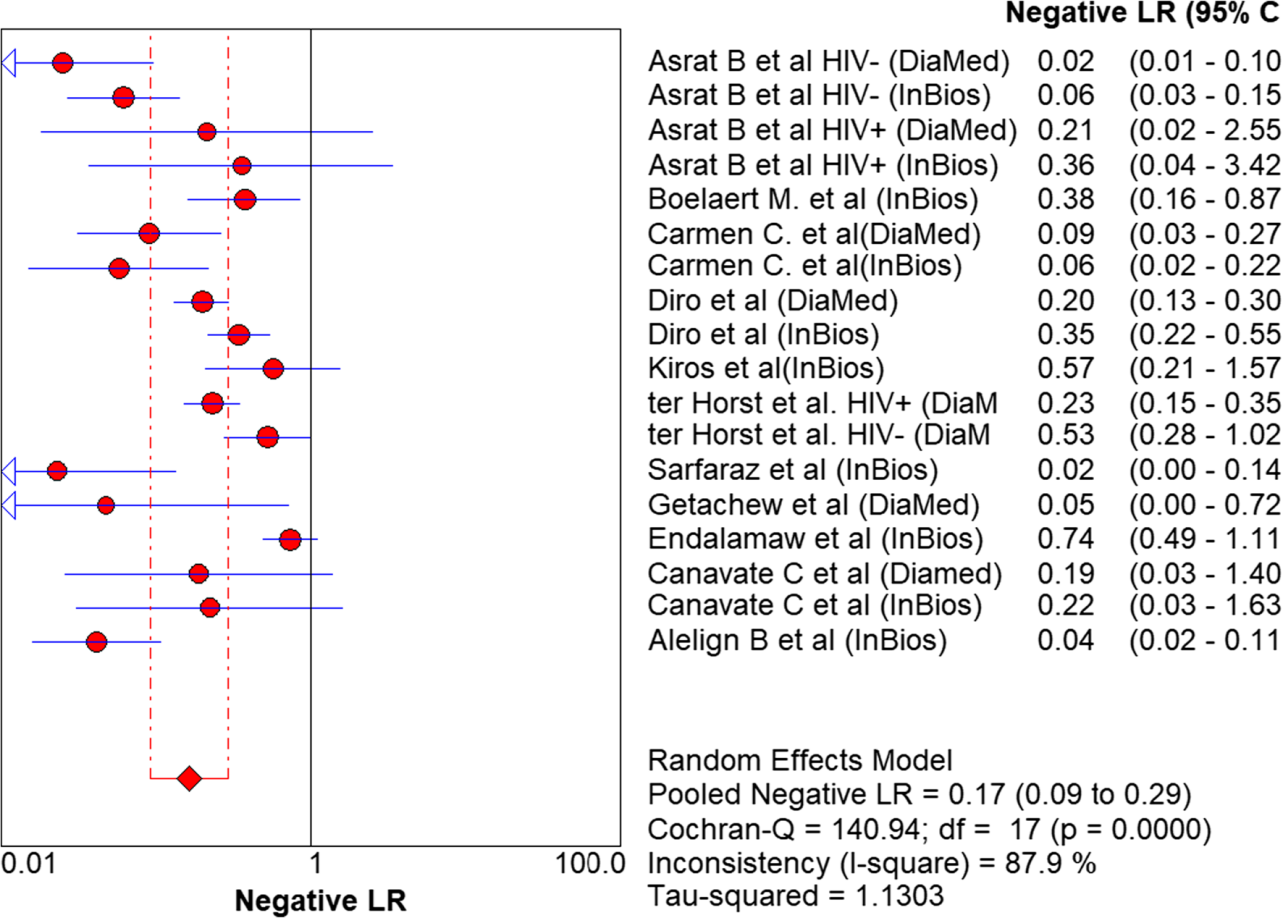
Estimates of negative likelihood ratio of rK39 for the diagnosis of VL in Ethiopian studies

Similarly, the negative likelihood ratio was also determined: 0.17 (95% CI 0.09% to 0.92%, (Fig. 5).

### Performance of rk39 among HIV seropositive individuals

In this meta-analysis, the sensitivity and specificity of rk39 among the HIV-positive and negative participants were determined. In HIV negatives, the sensitivity and specificity of the rk39 were 86.0% (95% CI 81.0% to 91.0%) and 97.0% (95% CI 94.0% to 99.0%) respectively. The sensitivity and specificity of rk39 among HIV positive VL patients were 82.0% (95% CI 76.0% to 87.0%) and 67.0% (22.0% to 96%) respectively.

**Fig. 6:**
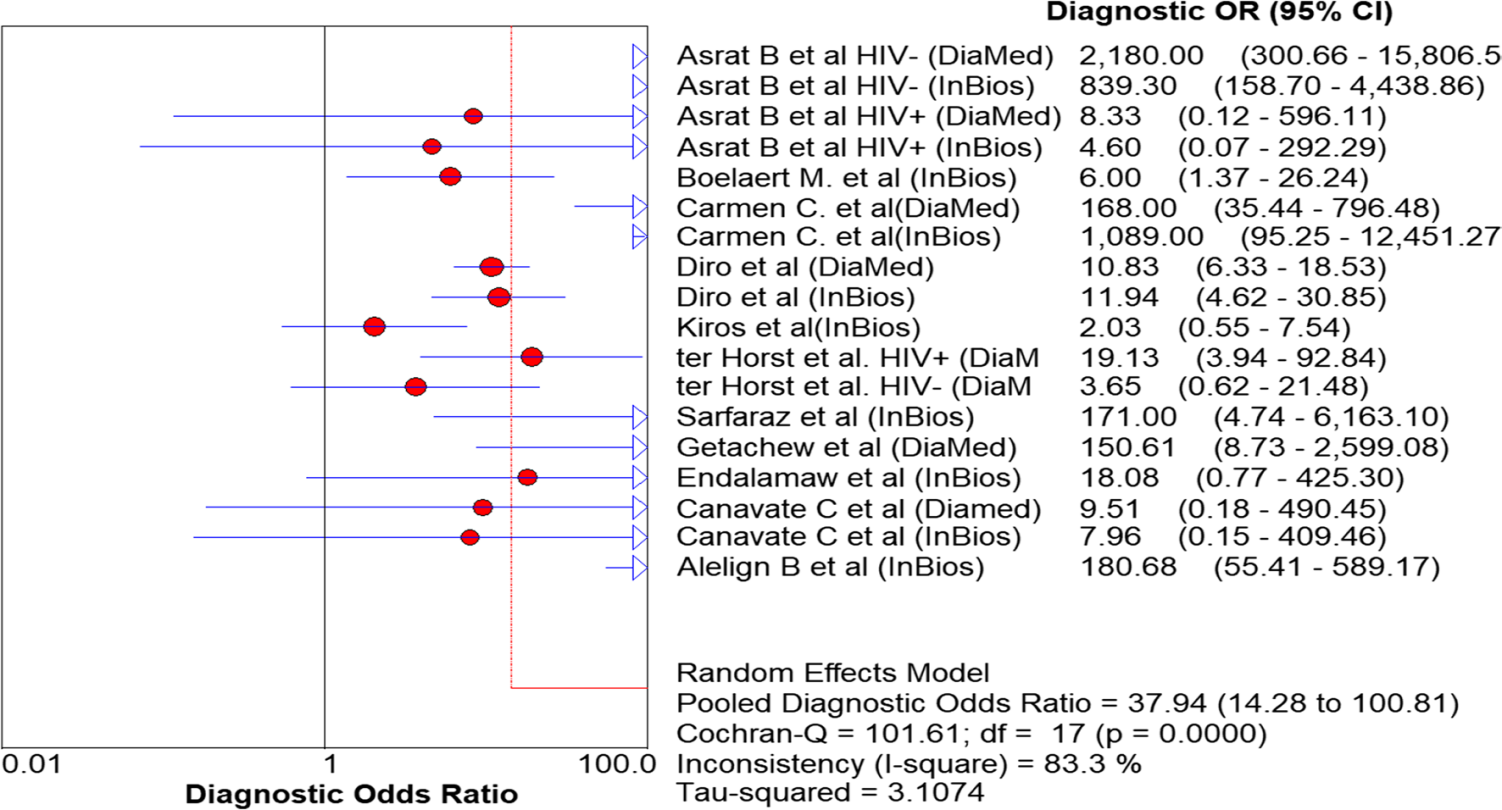
Diagnostic Odds Ratio of rk39 to diagnose VL in Ethiopia

The diagnostic odds ratio (DOR) is an important single indicator that explained the diagnostic capability of a test to correctly differentiate patients from the non-patients. The DOR was: 37.94 (95% CI 14.28 to 100.81), Fig. 6. However, when the two extreme outliers, Kiros et al. [20] and Endalamaw et al. [9], were excluded, the DOR is raised to 49.59 (17.79 to 136.26).

**Fig. 7:**
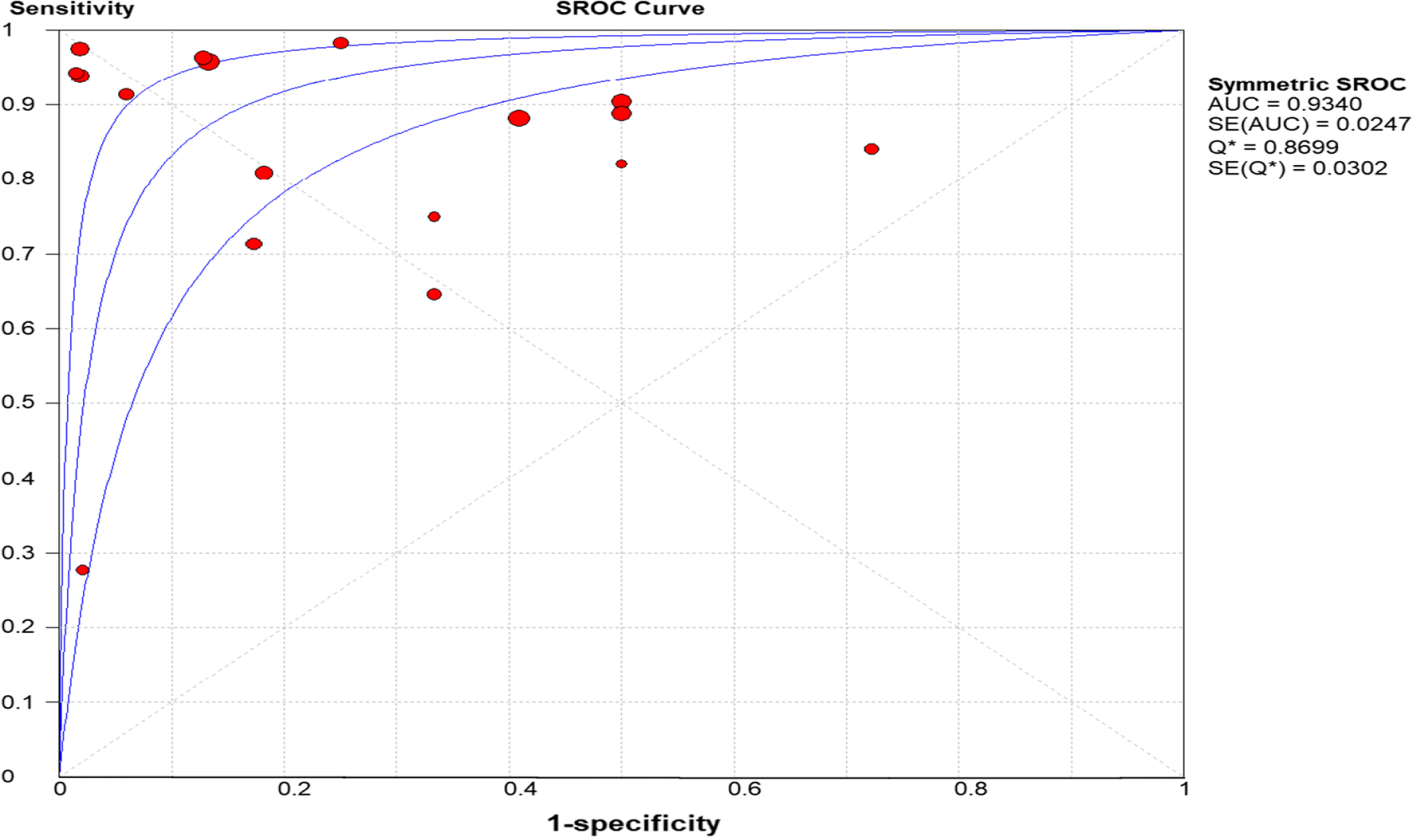
summary ROC of rk39 for the diagnosis of VL in Ethiopia

The SROC curve analysis is one of the valuable parameters to classify an individual into disease and without the disease [30]. In this meta-analysis, the SROC curve was generated and the area under the curve (AUC) was 93.4, Fig. 7. As part of heterogeneity testing in the SROC curve, we visually inspect the curve for the presence of shoulder arm pattern at the upper left corner and the pattern was observed which representing the existence of heterogeneity. According to Cochran's handbook guide for meta-analysis of diagnostic test accuracy, the currently available methods such as funnel plot are not appropriate to examine the presence of publication bias [31]. Therefore, we only determined Egger’s test *P-value* to examine whether there is a significant publication bias or not. In our analysis, Egger’s test yielded *P* = *0.014,* referring publication bias was statistically significant.

## Discussion

In this systematic review, we observed a very wide range of sensitivities and specificities of rk39 RDT among each study (27.8% to 98.3% and 27.8% to 98.5%) and from the pooled estimates (88.0% and 84.0%) respectively. The pooled sensitivity of the rk39 RDT was lower (88.0%) compared to the Indian sub-continent (97%) and the global sensitivity (91.9%) [32], and a little higher than the pooled sensitivity of the east African studies (85.3%). The variation possibly is explained by the commercial brand of rk39 RDT and the reference test used. More importantly, the sensitivity of the kit in the northern part of Ethiopia, a region with the highest VL burden, is lower (86.0%) compared to the other parts of Ethiopia (100.0%). The principle of the rk39 RDT is based on the detection of anti-leishmania antibodies in the patient’s serum, which persists for months and even for years after a patient recovered from the disease. In addition, anti-leishmania antibodies can be produced in asymptomatic and sub-clinical patients as well [22], which potentially reduced the diagnostic accuracy in endemic areas. The commercial brand of rk39 RDT, types of reference tests, and the presence of other comorbidities like HIV can lower the performance. Genetic diversity of rK39 gene sequences of *L. donovani* strains between East African and Indian or maybe due to population differences between the continents can be the cause for the performance disparities. Molecular characterization of the rK39 kinesin repeat sequences of *L. donovani* strains from East Africa demonstrated a clear divergence from the Southeast Asian strains, manifested by a variation in drug susceptibility patterns: Indian strains were sensitive to a certain drug whereas East Africa strains were resistant [22].

More interestingly, the northern and north-western strains are similar to the Sudanese strains whereas the southern strains are similar to the Kenyan strain [33–35], which perhaps, revealed the reason for performance disparities within Ethiopia. The major challenge of meta-analysis of diagnostic test accuracy in dealing with heterogeneity. In the present study, a considerable level of heterogeneity (I^2^ > 75) was determined. To assess the source of heterogeneity, subgroup analysis using a different type of reference test and commercial brand of rk39 was performed based. Accordingly, using different types of reference tests was found as source heterogeneity while variation in commercial Brand of rk39 RDT was not. Publication bias is one of the essential parameters to be determined during meta-analysis, especially in interventional studies. However, these methods are not appropriate for meta-analysis of diagnostic tests [31]. As a result, we only determined the Egger’s test p-values and observed a significant publication bias, *p* = *0.014*.

To include gray literature and unpublished manuscripts, searching specific Ethiopian University libraries and personal communications with individuals working at various research institutions in Ethiopia were made. Besides, we also checked the references of the included studies to increase the chance of getting more articles. We did not have any language and time restrictions, though we know studies done in Ethiopia are all in English.

The sensitivity and specificity of rk39 RDT were higher (90.7% and 90.2%) in studies that used serology than microscopy and/or NNN culture (84.4% and 74.1%) and PCR (89.8% and 50.0%) as reference tests. The possible explanation could be *L. donovani* complex can non-specifically activate B cells to produce cross-reactive antibodies that can affect the specificity of the test [36]. The specificity of rk39 among Human Immunodeficiency Virus (HIV) negative was higher (97.0%) than HIV positive (66.0%) VL patients. However, the sensitivity of HIV-positive (82.0%) and negative VL (86.0%) patients almost remained similar. HIV, known to deplete T cells, induces immune tolerance and lowering the renewal of the T-cell repertoire which leads to exhaustion of B cells response [37], which justifies the rk39 RDT performance disparities observed in this analysis.

Diagnostic odds ratio (DOR), which is not affected by disease prevalence, is an important single quantitative parameter that revealed the tests' ability to classify the individuals into diseased and not diseased [38]. In the present meta-analysis, the DOR of rk39 RDT to diagnose VL was 37.9 and therefore the odds of VL patients having a positive rk39 test result is approximately 38 times higher than those individuals without the disease. The likelihood ratio is also another essential indicator for the diagnostic test to assess how likely the VL patients have a positive diagnostic result [39]. Likelihood ratios range from zero to infinity, so the higher the value, the more likely the patient to have the disease. In the present meta-analysis, the positive likelihood ratio was 5.12 and hence the positive test result occurs 5.12 times more frequently in VL patients than the non-VL patients. Similarly, the negative likelihood ratio was 0.17 and hence, rk39 RDT negative test result was 1/0.17 = 5.9 times less frequent in VL patients than the non-VL patients. Moreover, another essential indicator of the performance of a diagnostic test is the SROC curve, which categorized patients into VL and non-VL, which is expressed by the AUC. The AUC has different scales; 0.9 to 1.0 = excellent, 0.8 to 0.9 = good, 0.7 to 0.8 = fair, and < 0.5 have no diagnostic value. Therefore, in the present meta-analysis, the AUC is 0.93 and hence, according to the result, rk39 is an excellent alternative diagnostic test for VL in endemic remote areas, Fig. 5.

## Limitation of the study

The major limitation of this meta-analysis was the individual studies employed diverse types of reference tests which affects the pooled sensitivity and specificity of the test. Apart from this, the rk39 RDT has certain inherent limitations as the test is based on anti-Leishmania antibodies, which can remain in the serum for a long time even after the parasite has cleared. On top of this, we did not perform meta-regression because the software did not support it.

## Conclusions

Referring to our result, rk39 considered an essential rapid diagnostic test for VL diagnosis. Besides the diagnostic accuracy, the features such as easy to perform, quick (10–20 min), cheap, equipment-free, electric and cold chain free, and result reproducibility, rk39 RDT is advisable to remains in practice as a diagnostic test at least in the remote VL endemic localities of Ethiopia till a better test will come.

## Data Availability

Not applicable.
